# Pre-pregnancy BMI, gestational weight gain and risk of preeclampsia: a birth cohort study in Lanzhou, China

**DOI:** 10.1186/s12884-017-1567-2

**Published:** 2017-12-01

**Authors:** Yawen Shao, Jie Qiu, Huang Huang, Baohong Mao, Wei Dai, Xiaochun He, Hongmei Cui, Xiaojuan Lin, Ling Lv, Dennis Wang, Zhongfeng Tang, Sijuan Xu, Nan Zhao, Min Zhou, Xiaoying Xu, Weitao Qiu, Qing Liu, Yawei Zhang

**Affiliations:** 1 Gansu Provincial Maternity and Child Care Hospital, 143 North Road, Qilihe District, Lanzhou, Gansu Province 730050 China; 2 Yale University School of Public Health, 60 College Street, New Haven, CT 06520 USA; 3 Yale School of Medicine, New Haven, CT USA

**Keywords:** Pre-pregnancy BMI, Gestational weight gain, Preeclampsia, China, Birth cohort

## Abstract

**Background:**

To evaluate the independent and joint effects of maternal pre-pregnancy BMI and gestational weight gain (GWG) on the risk of preeclampsia and its subtypes.

**Methods:**

A birth cohort study was conducted from 2010 to 2012 in Lanzhou, China. Three hundred fourty seven pregnant women with preeclampsia and 9516 normotensive women at Gansu Provincial Maternity and Child Care Hospital were included in the present study. Unconditional logistic regression models were used to evaluate the associations between pre-pregnancy BMI, GWG, and risk of preeclampsia and its subtypes.

**Results:**

Compared to women with normal pre-pregnancy BMI, those who were overweight/obese had an increased risk of preeclampsia (OR = 1.81; 95%CI: 1.37–2.39). Women with excessive GWG had an increased risk of preeclampsia (OR = 2.28; 95%CI: 1.70–3.05) compared to women with adequate GWG. The observed increased risk was similar for mild-, severe- and late-onset preeclampsia. No association was found for early-onset preeclampsia. Overweight/obese women with excessive GWG had the highest risk of developing preeclampsia compared to normal weight women with no excessive weight gain (OR = 3.78; 95%CI: 2.65–5.41).

**Conclusions:**

Our results suggested that pre-pregnancy BMI and GWG are independent risk factors for preeclampsia and that the risk might vary by preeclampsia subtypes. Our study also proposed a potential synergistic effect of pre-pregnancy BMI and GWG that warrants further investigation.

## Background

Preeclampsia is defined as the development of hypertension and proteinuria after 20 weeks of gestation [1]. It affects up to 8% of all pregnancies worldwide and increases morbidity and mortality rates among both mothers and infants [2, 3]. Preeclampsia is the leading cause of prematurity and fetal growth restriction [4, 5]. The mortality rate among babies born to mothers with preeclampsia is five times higher than that among babies born to healthy mothers [6]. Preeclampsia is also the second leading cause of pregnancy-related intensive care unit admissions after obstetric hemorrhage [7]. Furthermore, preeclampsia is associated with an elevated risk of cardiovascular disease later in life [8, 9].

Given the known and potential adverse consequences of preeclampsia, an understanding of the risk factors of this condition is warranted. A wide range of pregnancy-specific characteristics (e.g. parity, placental factors, multi-fetal gestation, and excessive weight gain during pregnancy) and pre-existing maternal features (e.g. age, race, pre-pregnancy overweight or obesity, pre-pregnancy diabetes, chronic hypertension etc.) are considered to be associated with preeclampsia [10]. Pre-pregnancy BMI and gestational weight gain (GWG) are two modifiable risk factors [11–13]. Both pre-pregnancy BMI and GWG may increase oxidative stress levels, stimulate a systemic inflammatory response, and accelerate damage to vascular endothelial cells, resulting in preeclampsia [14, 15]. Studies from different populations have consistently reported that elevated pre-pregnancy BMI is associated with an increased risk of preeclampsia [16–38]. However, the relationship between GWG and preeclampsia is still inconclusive, with some studies suggesting a positive association [9, 30, 39–49] and others reporting no association [11, 21, 50, 51]. Few studies have examined the relationship between pre-pregnancy BMI, GWG and the risk of preeclampsia by different subtypes [14, 30, 43, 52, 53]. Even fewer studies have investigated the joint effect of pre-pregnancy BMI and GWG on preeclampsia and its subtypes. Here, we analyzed data from a birth cohort study conducted in Lanzhou, China [54, 55] to evaluate the independent and joint effects of maternal pre-pregnancy BMI and GWG on the risk of preeclampsia and its various subtypes.

## Methods

A birth cohort study was carried out from 2010 to 2012 at Gansu Provincial Maternity and Child Care Hospital, the largest hospital of its kind in Lanzhou, China. Eligible study participants were pregnant women who came to the hospital for delivery with gestational age ≥ 20 weeks, who had no history of mental illness, and who were 18 years or older. A total of 14,359 eligible women were identified and invited to participate. Of those, 3712 refused to participate and 105 did not complete in-person interviews, yielding 10,542 (73.4%) women with completed interviews. Upon obtaining written consent, a standardized and structured questionnaire was used to collect information on demographic factors, reproductive and medical history, smoking and alcohol consumption, occupational and residential history, physical activity, and diet. Information on pregnancy complications and birth outcomes were abstracted from medical records. After excluding women with pre-existing chronic hypertension before pregnancy and missing values of pre-pregnancy BMI or GWG, the final sample size was 9863. Among these women, 347 were diagnosed with preeclampsia. All study procedures were approved by the Human Investigation Committees at the Gansu Provincial Maternity and Child Care Hospital and Yale University. Additional detailed information on the cohort has previously been published [54, 55].

Preeclampsia was defined as hypertension (two separate blood pressure readings ≥ 140/90 mmHg taken at least 6 h apart) and proteinuria (≥ 1+ on dipstick test in two urine samples or ≥ 300 mg of protein in a 24 h urine sample) after 20 weeks of gestation. Preeclampsia was further subcategorized as mild preeclampsia (M-PE) and severe preeclampsia (S-PE), as well as early-onset preeclampsia (EOPE) and late-onset (LOPE) [54]. M-PE was defined as raised blood pressure (≥ 140/90 mmHg and <160/110 mmHg) and proteinuria (≥ 1+ and <2+ on dipstick test in two urine samples) without symptoms of severity. S-PE was defined as raised blood pressure (≥ 160/110 mmHg) and proteinuria (≥ 2+ on dipstick test in two urine samples) with additional symptoms of severity such as headache, blurred vision, epigastric pain, decreased urine output, and decreased or absent fetal kick. Women with EOPE had preeclampsia before 34 weeks of gestation, while those with LOPE had preeclampsia at or after 34 weeks of gestation.

Pre-pregnancy weight was self-reported during the first prenatal care visit. Pre-pregnancy BMI was calculated as weight (kg) divided by the square of height (m), and then subcategorized as underweight (BMI < 18.5 kg/m^2^), normal weight (18.5 kg/m^2^ ≤ BMI < 24 kg/m^2^), and overweight (BMI≥24 kg/m^2^) groups. Since East Asians have a higher body fat percentage than Caucasians [56], the BMI cutoffs for overweight and obesity differ between Eastern and Western populations. The standards used in this study were established by the Working Group of Obesity in China [57]. As only a small number of women were obese, overweight and obese women were combined to increase statistical power.

Gestational weight gain (GWG) in kg was calculated by subtracting pre-pregnancy weight from maternal weight at delivery. Since there were no official recommendations specific to the Chinese population, GWG was categorized based on the US Institute of Medicine (IOM) GWG Guidelines 2009 [58]. Adequate weight gain was defined as 12.5–18.0 kg, 11.5–16.0 kg, and 7.0–11.5 kg for underweight, normal weight, and overweight women, respectively.

Differences in selected characteristics between women with preeclampsia and normotensive women were evaluated using Chi-square tests or Fisher’s exact tests if necessary. Unconditional logistic regression was used to determine odds ratios (OR) and 95% confidence intervals (CI) for the associations between pre-pregnancy BMI, GWG, and the risk of preeclampsia and its subtypes. Confounding factors including maternal age, maternal employment during pregnancy, monthly household income, maternal education level, parity, twin status, newborn gender, and family history of hypertension were adjusted for in the unconditional logistic regression models. All statistical tests were two-sided. Analyses were performed using SAS 9.3 (SAS Institute, Inc., Cary, NC, USA).

## Results

A total of 9863 women were included in the final analysis of which 347 (3.52%) were diagnosed with preeclampsia. Among those with preeclampsia, 206 (59.4%) had S-PE and 141 (40.6%) had M-PE, while 304 (87.6%) had LOPE, and 43 (12.4%) had EOPE. The prevalence of pre-pregnancy underweight, normal weight and overweight (including obesity) were 21.33%, 67.92%, and 10.75%, respectively.

Table 1 shows general characteristics of the study population. Compared to normotensive women, women with preeclampsia were more likely to be older, unemployed, less educated, multiparous, pregnant with a female fetus or multiple fetuses, had lower monthly household income and a family history of hypertension. The distributions of maternal diabetes, smoking (active and passive) during pregnancy, alcohol consumption during pregnancy, and physical activity during pregnancy were similar between women with and without preeclampsia.

**Table 1:** Distribution of Selected Characteristics of the Study Population

Characteristics	All participants		Preeclampsia		*P*-value^a^
	n	(%)	n	(%)	
All	9863	100	347	3.5	
Maternal age					<0.001
< 25y	4780	48.5	131	2.7	
25-29y	1530	15.5	52	3.4	
≥ 30y	3553	36.0	164	4.6	
Employment status					0.0012
Yes	5180	52.5	151	2.9	
Not during pregnancy	1524	15.5	56	3.7	
Never	3159	32.0	140	4.4	
Monthly income (RMB)					<0.0001
< 3000	4995	50.6	227	4.5	
≥ 3000	3998	40.5	84	2.1	
Education level					<0.0001
≥ college	3734	37.9	83	2.2	
< college	5878	59.6	250	4.3	
Parity					0.0035
Multifarious	2679	27.2	118	4.4	
Primiparous	7184	72.8	229	3.2	
Newborn gender					0.0073
Male	5200	52.7	159	3.1	
Female	4633	47.0	188	4.1	
Twin					<0.0001
Yes	284	2.9	58	20.4	
No	9579	97.1	289	3.0	
Family history of hypertension					<0.0001
Yes	1510	15.3	91	6.0	
No	8353	84.7	256	3.1	
Pre-pregnancy BMI^b^					
Normal weight	6699	67.9	221	3.3	<0.0001
Underweight	2104	21.3	46	2.2	
Overweight	1060	10.7	80	7.5	
Gestational weight gain(GWG)					<0.0001
Inadequate	1323	13.4	33	2.5	
Adequate	3279	33.2	62	1.9	
Excessive	5261	53.3	252	4.8	
Maternal diabetes					
Yes	97	1.0	7	7.2	0.085^*^
No	9766	99.0	340	3.5	
Smoking (passive and active) during pregnancy					0.323
Yes	1928	19.5	75	3.9	
No	7935	80.5	272	3.4	
Alcohol consumption during pregnancy					1^*^
Yes	17	0.2	0		
No	9846	99.8	347	3.5	
Physical activity during pregnancy					0.796
Yes	8250	83.6	292	3.5	
No	1613	16.4	55	3.4	

^a^The analysis did not account for missing data. For variable Monthly income (RMB), data was missing for 870 participants, for variable Education level, data was missing for 251 participants, for variable Newborn gender, data was missing for 30 participants

^b^Weight(kg) / height^2^ (m^2^)

^*^Fisher’s exact test, for all other variables Chi-square test

Pre-pregnancy overweight or obesity was associated with an increased risk of preeclampsia (OR = 1.81, 95%CI: 1.37–2.39), M-PE (OR = 1.76, 95%CI: 1.14–2.71), S-PE (OR = 1.79, 95%CI: 1.26–2.54), and LOPE (OR = 1.79, 95%CI: 1.33–2.41) compared to normal weight (Table 2). Underweight was associated with a reduced risk of S-PE (OR = 0.60, 95%CI: 0.38–0.95) compared to normal weight. Compared to women with adequate GWG, women with excessive GWG had more than a two-fold increased risk of preeclampsia (OR = 2.28, 95%CI: 1.70–3.05), M-PE (OR = 2.79, 95%CI: 1.74–4.47), S-PE (OR = 2.03, 95%CI: 1.41–2.92), and LOPE (OR = 2.53, 95%CI: 1.84–3.48). Inadequate GWG was not associated with the risk of preeclampsia and its subtypes. We further analyzed GWG using the quartiles of GWG among normotensive women. Compared to the lowest GWG quartile, the highest quartile was associated with an increased risk of preeclampsia (OR = 2.59, 95%CI: 1.90–3.53), M-PE (OR = 3.55, 95%CI: 2.13–5.92), S-PE (OR = 2.17, 95%CI: 1.48–3.19), and LOPE (OR = 2.95, 95%CI: 2.10–4.13). The second highest GWG quartile was associated with an increased risk of preeclampsia (OR = 1.66, 95%CI: 1.18–2.33), M-PE (OR = 2.17, 95%CI: 1.25–3.78), and LOPE (OR = 1.74, 95%CI: 1.20–2.52). A significant P trend was observed for preeclampsia, M-PE, S-PE, and LOPE. We also found a decreased risk of EOPE associated with the second GWG quartile (OR = 0.30, 95%CI: 0.10–0.92), but this association was based on four exposed cases.

**Table 2:** Associations of Pre-pregnancy BMI, Total GWG with the Risk of Preeclampsia and Subtypes (*N* = 9863)^a,b^

	Control	PE		M-PE		S-PE		EOPE		LOPE	
	*n* = 9516	Cases *n* = 347	OR (95% CI)	Cases *n* = 141	OR (95% CI)	Case *n* = 206	OR (95% CI)	Case n = 43	OR (95% CI)	Case *n* = 304	OR (95% CI)
Pre-pregnancy BMI (kg/m^2^)											
Normal weight	6478	221	1.00	86	1.00	135	1.00	29	1.00	192	1.00
Underweight	2058	46	0.76 (0.54–1.06)	24	1.00 (0.62–1.59)	22	0.60 (0.38–0.95)	4	0.49 (0.17–1.42)	42	0.80 (0.57–1.14)
Overweight/obese	980	80	1.81 (1.37–2.39)	31	1.76 (1.14–2.71)	49	1.79 (1.26–2.54)	10	1.84 (0.87–3.90)	70	1.79 (1.33–2.41)
Total GWG (kg) by IOM Guidelines											
Adequate	3217	62	1.00	22	1.00	40	1.00	12	1.00	50	1.00
Inadequate	1290	33	1.27 (0.82–1.96)	11	1.24 (0.59–2.57)	22	1.29 (0.75–2.19)	5	0.99 (0.34–2.85)	28	1.34 (0.83–2.15)
Excessive	5009	252	2.28 (1.70–3.05)	108	2.79 (1.74–4.47)	144	2.03 (1.41–2.92)	26	1.23 (0.61–2.50)	226	2.53 (1.84–3.48)
Total GWG (kg) Change by quartile^c^											
GWG ≤ 13.5	2500	68	1.00	21	1.00	47	1.00	14	1.00	54	1.00
13.5 < GWG ≤ 17	2711	55	0.85 (0.59–1.23)	21	1.03 (0.56–1.89)	34	0.78 (0.49–1.22)	4	0.30 (0.10–0.92)	51	0.99 (0.67–1.47)
17 < GWG ≤ 20	2101	81	1.66 (1.18–2.33)	35	2.17 (1.25–3.78)	46	1.44 (0.94–2.20)	13	1.35 (0.62–2.93)	68	1.74 (1.20–2.52)
20 < GWG	2204	143	2.59 (1.90–3.53)	64	3.55 (2.13–5.92)	79	2.17 (1.48–3.19)	12	1.17 (0.53–2.59)	131	2.95 (2.10–4.13)
P_trend_			<0.0001		<0.0001		<0.0001		0.357		<0.0001

^a^Pre-pregnancy BMI, total GWG and total GWG change were evaluated in separate models

^b^Adjusted for maternal age, maternal employment during pregnancy, education level, monthly household income, newborn gender, parity, twin status, family history of hypertension; pre-pregnancy BMI and total GWG were mutually adjusted

^c^Distribution of GWG based on quartile

Joint effects between pre-pregnancy BMI and GWG on the risk of preeclampsia and its subtypes are presented in Table 3. Women with both pre-pregnancy overweight (including obesity) and excessive GWG had the highest risk of preeclampsia (OR = 3.78, 95%CI: 2.65–5.41), M-PE (OR = 4.82, 95%CI: 2.71–8.59), S-PE (OR = 3.22, 95%CI: 2.06–5.03), and LOPE (OR = 4.11, 95%CI: 2.81–6.03), although there was no statistically significant interaction between pre-pregnancy BMI and GWG (P_interaction_ > 0.05).

**Table 3:** Joint Effects of Pre-pregnancy Maternal BMI and GWG on Preeclampsia and Subtypes in Different Groups (N=9863)

Pre-pregnancy BMI	Weight Gain During Pregnancy (GWG) by IOM Guidelines						
	Not Excessive			Excessive^a^			P_interaction_
	Cases	Controls	OR^b^ (95%CI)	Cases	Controls	OR^b^ (95% CI)	
Preeclampsia							0.69
Underweight	19	1234	0.75 (0.44-1.27)	27	824	1.65 (1.03-2.64)	
Normal weight	65	3054	1.00	156	3424	2.16 (1.60-2.92)	
Overweight/obese	11	219	2.10 (1.08-4.06)	69	761	3.78 (2.65-5.41)	
M-PE							0.87
Underweight	10	1234	1.16 (0.54-2.49)	14	824	2.42 (1.21-4.84)	
Normal weight	21	3054	1.00	65	3424	2.68 (1.62-4.42)	
Overweight/obese	2	219	1.22 (0.28-5.27)	29	761	4.82 (2.71-8.59)	
S-PE							0.47
Underweight	9	1234	0.55 (0.26-1.13)	13	824	1.26 (0.66-2.38)	
Normal weight	44	3054	1.00	91	3424	1.94 (1.34-2.82)	
Overweight/obese	9	219	2.45 (1.17-5.13)	40	761	3.22 (2.06-5.03)	
EOPE							0.11
Underweight	2	1234	0.51 (0.11-2.31)	2	824	0.81 (0.18-3.73)	
Normal weight	11	3054	1.00	18	3424	1.61 (0.75-3.45)	
Overweight/obese	4	219	4.48 (1.40-14.31)	6	761	1.97 (0.72-5.37)	
LOPE							0.80
Underweight	17	1234	0.80 (0.46-1.40)	25	824	1.81 (1.10-2.97)	
Normal weight	54	3054	1.00	138	3424	2.27 (1.64-3.15)	
Overweight/obese	7	219	1.59 (0.71-3.56)	63	761	4.11 (2.81-6.03)	

^a^Excessive weight gain: weight gain above the IOM recommendations, defined as weight gain during pregnancy over 18 kg, 16 kg, and 11.5 kg for underweight, normal weight, and overweight women, respectively

^b^Adjusted for maternal age, maternal employment during pregnancy, education level, monthly household income, newborn gender, parity, twin status, family history of hypertension

## Discussion

Our study supported that pre-pregnancy overweight and excessive GWG were independently associated with an increased risk of preeclampsia and that the risk might vary by its clinical subtypes. Higher BMI is associated with a risk of preeclampsia in a dose-dependent manner. The present study also found that the positive association between pre-pregnancy BMI and preeclampsia was similar for S-PE and M-PE, but different for LOPE and EOPE, as pre-pregnancy BMI had a positive association with LOPE but no association with EOPE.

Excessive GWG is associated with an increased risk of preeclampsia. Our study also found that the association between GWG and preeclampsia varied by subtype. We observed an increased risk of M-PE, S-PE, and LOPE, but not EOPE, associated with excessive GWG.

In our study, the highest risk for preeclampsia, S-PE, M-PE, and LOPE was observed among women who were overweight/obese and had an excessive GWG, although the interactions between pre-pregnancy BMI and GWG were not statistically significant. A potential synergistic effect between pre-pregnancy BMI and GWG warrants further investigation.

The classic concept suggests that preeclampsia is a two-stage disorder [59, 60]. The first stage involves abnormal implantation, including shallow trophoblastic invasion and insufficient spiral artery remodeling or other pathological disorders leading to decreased placental perfusion. During the second stage, maternal systemic inflammatory response and oxidative stress converge to alter vascular endothelium function, ultimately leading to multi-organ damage [10, 59–62]. The metabolic and biochemical disturbances associated with overweight and obesity may provide the maternal milieu associated with the second stage of preeclampsia [33]. Overweight/obesity, which is considered a chronic inflammatory condition, increases the levels of plasma C-reactive protein and certain inflammatory cytokines [63–65]. This leads to a systemic inflammatory response, resulting in an increase in neutrophils that release toxic compounds (i.e. reactive oxygen species and myeloperoxidase), capable of attacking and destroying vascular endothelium cell integrity. This mechanism ultimately causes the clinical symptoms of preeclampsia [66].

The association between higher BMI and risk of preeclampsia reported in our study is consistent with that of previous studies based on both Western populations [17–21, 23, 26, 28–30, 32–36, 38], and Asian populations [16, 22, 24, 25, 27, 31, 37, 67, 68]. Among the few previous studies that investigated the association between pre-pregnancy BMI and preeclampsia subtypes [14, 19, 30, 38], their results suggested that overweight/obesity before pregnancy increased the risk of S-PE [14, 19], M-PE [30], LOPE [30, 38], but not EOPE [14, 19, 30, 38]. This finding was supported in our study. The lack of a significant association between pre-pregnancy BMI and EOPE in our study could be due to the small number of EOPE cases (*n* = 43). The consistency of this finding with others suggest that EOPE and LOPE are two different diseases associated with different biochemical markers, risk factors, clinical features, and hemodynamic states [69]. For example, EOPE is typically associated with fetal growth restriction, reduction in placental volume [69], abnormal uterine and umbilical artery Doppler evaluation [70], as well as adverse maternal and neonatal outcomes — maternal mortality is approximately 20-folds higher for preeclampsia cases that manifest at less than 32 weeks’ gestation compared to those that occur at term [71]. In contrast, LOPE often involves normal fetal growth, larger placental volume, normal birth weight and favorable maternal and neonatal outcomes [72].

Our results supported those of previous studies showing that excessive GWG is associated with an increased risk of preeclampsia [9, 30, 39–49], and contrary to those of other studies [11, 21, 50, 51]. Differences in results could be due to the heterogeneity of study designs and methods. Some studies [9, 11, 21, 30, 39, 40, 44, 45] adopted 2009 IOM GWG Guidelines to classify GWG according to pre-pregnancy BMI categories as defined by the WHO, others [41, 42, 48, 50] used the 1990 IOM GWG Guidelines to categorize GWG according to pre-pregnancy BMI categories based on Metropolitan Life Insurance Company’s weight-for-height standards, and the rest [43, 46, 47, 49] did not use the US IOM GWG Guidelines. In addition to differences in GWG categorization, variations in study population (different ethnic/race distribution) and sources of GWG data (self-reported vs medical record) might also contribute to the inconsistency of the study results.

Previous studies suggested that different preeclampsia subtypes may have different features [69], potentially accounting for varying synergistic effects between pre-pregnancy BMI and GWG with different preeclampsia subtypes. However, studies on synergistic effect between pre-pregnancy BMI and GWG with preeclampsia are scarce: only two previous studies [13, 49] evaluated the combined effects of pre-pregnancy BMI and GWG on preeclampsia. Both of the studies were based on Western populations, and neither of them examined potential associations with different preeclampsia subtypes. To address the literature gap, our study sought to analyze these joined effects on Asian populations. According to our results, women who were overweight/obese before pregnancy and had an excessive GWG had the highest risk for preeclampsia, S-PE, M-PE, and LOPE. Interestingly, the interaction between pre-pregnancy BMI and GWG was not statistically significant. The potential combined effects of pre-pregnancy BMI and GWG on different preeclampsia subtypes require further investigation.

There were several strengths and limitations to our study. Detailed information on demographic factors, medical histories, and lifestyle factors allowed us to control for important confounding factors. Diagnoses of preeclampsia and its subtypes based on medical records rather than self-reports, minimized potential disease misclassification. In terms of pre-pregnancy weight, such data was self-reported, potentially resulting in unavoidable recall bias. Based on previous literature, pre-gravid overweight/obese women are more likely to underreport pre-pregnancy weight than normal weight women [73]. As information on GWG by trimester was unavailable, we were not able to distinguish between weight gain from adiposity (early weight gain) and that from edema (later weight gain). Previous studies have shown that greater weight gain in early pregnancy led to an elevated risk of future gestational hypertension [74, 75], proposing that adipose tissue rather than edema is part of the etiology of pregnancy-induced hypertension. Further investigations focusing on weight gain trajectory during pregnancy and disease progression are necessary to better understand the effect of pre-pregnancy BMI and GWG on preeclampsia and its subtypes.

## Conclusions

In conclusion, our study results support that pre-pregnancy overweight (including obesity) and excessive GWG are independently associated with an increased risk of preeclampsia and the risk may vary by its clinical subtypes. A potential synergistic effect between pre-pregnancy BMI and GWG warrants further investigation. Consequently, future preventive strategies are needed to address pre-pregnancy overweight and obesity and to limit gestational weight gain in order to prevent preeclampsia.
